# The Use of Maleic Hydrazide for Effective Hybridization of *Setaria viridis*

**DOI:** 10.1371/journal.pone.0125092

**Published:** 2015-04-24

**Authors:** Govinda Rizal, Shanta Karki, Richard Garcia, Nikki Larazo, Michael Alcasid, William Paul Quick

**Affiliations:** 1 C4 Rice Center, International Rice Research Institute (IRRI), Los Baños, Laguna, the Philippines; 2 University of Sheffield, Sheffield, United Kingdom; National Institute of Plant Genome Research, INDIA

## Abstract

An efficient method for crossing green foxtail (*Setaria viridis*) is currently lacking. *S*. *viridis* is considered to be the new model plant for the study of C4 system in monocots and so an effective crossing protocol is urgently needed. *S*. *viridis* is a small grass with C4-NADP (ME) type of photosynthesis and has the advantage of having small genome of about 515 Mb, small plant stature, short life cycle, multiple tillers, and profuse seed set, and hence is an ideal model species for research. The objectives of this project were to develop efficient methods of emasculation and pollination, and to speed up generation advancement. We assessed the response of *S*. *viridis* flowers to hot water treatment (48°C) and to different concentrations of gibberellic acid, abscisic acid, maleic hydrazide (MH), and kinetin. We found that 500 μM of MH was effective in the emasculation of *S*. *viridis*, whilst still retaining the receptivity of the stigma to pollination. We also report effective ways to accelerate the breeding cycle of *S*. *viridis* for research through the germination of mature as well as immature seeds in optimized culture media. We believe these findings will be of great interest to researchers using *Setaria*.

## Introduction

Green foxtail (*Setaria viridis)* is one of the most widely used genetic systems for research on C_4_ photosynthesis and Panicoid grasses; in addition it is the ancestral wild relative of *S*. *italica*, an important cultivated millet [1, 2]. With the increased interest and funding for C_4_ photosynthesis research related to the wider aim of the introduction of C_4_ characteristics into C_3_ cereals to enhance yield, *S*. *viridis* has been widely used for basic research for the identification of genes related to C_4_ biochemistry and leaf anatomy [3–5]. It is also an emerging model system for C_4_ monocots, weeds, and biofuel research because of its short life cycle, small plant stature, small genome of about 515 Mb (2n = 18), simple cultivation requirement, and profuse seed set [6].

A significant number of the available literature on *Setaria* species focus on the optimization of its growth conditions, seedling establishment and germination [7–10]. However, reports on hybridization strategies are few [11, 12], and they are far from being standardized and efficient [5]. For genetic studies such as gene identification and population improvement, efficient hybridization techniques are necessary so that different types of populations can be developed. *S*. *viridis* has perfect hermaphrodite flowers with three stamens and a bifurcated pistil inside each small spikelet. It is self-pollinating and also cross-compatible with natural cross pollination rates of up to 4% [13]. The precise crossing of *S*. *viridis* for basic and applied research is limited by a few major barriers such as complex flowering pattern and long flowering period. Spikelets open during the cool hours of the day and anthesis is neither uniform nor follows a pattern [4]. It takes many days to complete flowering within a panicle. Manual emasculation is tedious and labor consuming. The spikelets are prone to shattering even with minor disturbances like shaking; hence manual emasculation is not preferred in large scale crossing programs. Several methods of emasculation have been reported in different crops which include plastic bag emasculation in sorghum and warm water emasculation in buckwheat [14, 15]. Even in a single crop like rice, several emasculation techniques are reported such as a mechanical method, a hot water method, manual emasculation, a suction method, alcohol treatment, cold treatment, genetic emasculation, and the use of gametocides [16–24]. However, efficient methods for emasculation of *S*. *viridis* are not available.

A range of chemicals are used as gametocides, like gibberellic acid (GA3), in different genotypes of German chamomile (*Matricaria recutita*) and in oil crops [25, 26]. Kalidasu et al. (2009) [27] reported an assessment of five gametocides including GA3 and maleic hydrazide (MH) in coriander flowers (*Coriandrum sativum*). However, there are no previously identified chemical gametocides suitable for the efficient emasculation of *S*.*viridis*. The use of such chemical gametocides could make emasculation of *S*. *viridis* much more efficient. This research, therefore, aimed to (1) identify an appropriate chemical for emasculation, optimize the method of emasculation, and cross-pollination; (2) confirm crosses through molecular methods; and (3) find means to accelerate the advancement of generations. We tested the effectiveness of different chemicals for emasculation and here, we report that MH was the most effective gametocide. We also report a simplified method of manual emasculation that is suitable in small experiments and when chemicals are not readily available. We also confirm the ability of *S*. *viridis* seeds (immature and mature) and F_1_ seeds to germinate in plant growth medium and we report the optimum condition that helps shorten the life cycle of *S*. *viridis*. These findings will greatly complement the efforts of researchers working on this C_4_ monocot.

## Materials and Methods

### Cultivation of plants

The seeds of *S*. *viridis* were obtained from North Central Regional Plant Introduction Station of the United States Department of Agriculture and were multiplied at IRRI, Philippines. *S*. *viridis* accessions A10.1 and PI 202407 were used for the development and optimization of emasculation and crossing techniques. The seedlings were grown in plastic cups or root trainers inside contained greenhouse conditions. Soil was mixed with fertilizers nitrogen, phosphorous and potassium (NPK, 1:1:1) at a rate of 10 g per 100 kg of soil. Seedlings were watered twice a day for the first week and once a day thereafter to maintain about 50% soil moisture content. Succession planting was scheduled to coincide with the flowering times of the different accessions.

### Chemical solution preparation and treatments

We prepared 100, 200 and 500 μM each of GA3, MH, abscisic acid (ABA), and kinetin (KNT). All four chemicals were dissolved in water at room temperature. In case of MH, the pH dropped to 4.5 which inhibited complete dissolution, so 1N NaOH was used to increase the pH to 7 to dissolve it completely. Ready to use solutions were stored at 4°C until used. Solutions were used within 5 days of preparation. For hot water treatment, a water bath with adjustable temperature was used. Water at room temperature was used as a control for the hot water treatment. Plants used as checks were trimmed and handled similarly but were not given any treatments.

### Chemical emasculation

**Fig 1:**
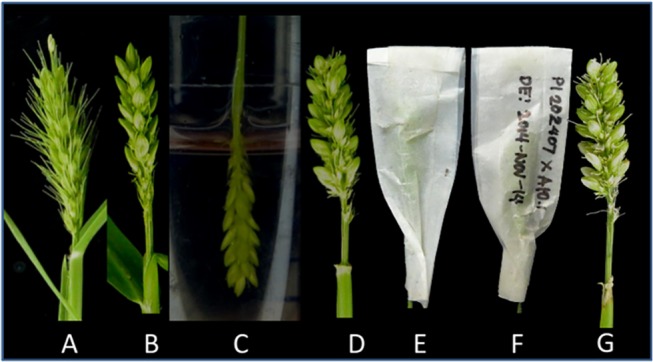
Emasculation of *Setaria viridis*. (A) Panicles that had just emerged completely out of the flag leaf sheath, such that the lowest spikelets of the panicles were between 0 and 2 cm from the collar of the flag leaf were selected. (B) The spikelets from tip and base of the panicle were trimmed; leaving approximately 50 florets and the bristles were also trimmed. (C) The panicles were completely immersed in the MH solution for 2 min, and excess liquid was gently wiped using tissue paper and (D) then the plant was left to stand until the panicles were dry. (E) After wiping off the solution and drying, the panicles were placed in glassine bags of appropriate sizes. (F) The glassine bag was labeled with the plant designation and emasculation date. Steps D and E were repeated on the same panicle for three consecutive mornings. (G) Panicle, 10 days after pollination.

We used plants with spikelets that had just emerged from their flag leaf sheath, when the lowest spikelets were between 0 and 2 cm from the uppermost collar of the flag leaf (Fig 1A). The selected panicles were trimmed from the top and spikelets from the base of the peduncle were carefully removed, leaving about 50 florets per panicle for chemical emasculation (Fig 1B). The bristles were trimmed off using sharp scissors (Fig 1B). For chemical emasculation, freshly prepared chemical solutions were used (S1 Protocol). The panicles were dipped completely into the solution for 2 min (Fig 1C) and wiped with tissue paper to dry the panicles and to prevent the solution from affecting other parts of the plants (Fig 1D). After drying, the panicles were placed inside glassine bags of appropriate sizes (Fig 1E). The glassine bags were labeled with name of plant and date of emasculation (Fig 1F). The panicles were dipped in the solution for 2 minutes, once a day in the morning (8:00 to 10:00 am) for three consecutive mornings.

For chemical emasculation using other chemicals and for hot water treatment, the same method was followed as above. In the hot water treatments, water temperatures were maintained at 44, 45, 46, 47, 48, 49, and 50°C. Water at room temperature and no treatment were used as controls.

### Manual emasculation

**Fig 2:**
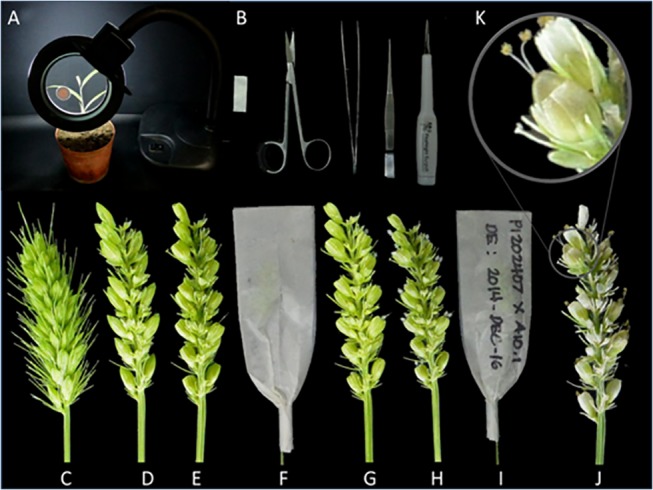
Manual emasculation of *Setaria viridis*. Equipment needed for manual emasculation included (A) a magnifying glass, (B) glassine bag, scissors, forceps and ear pick. (C) A panicle of correct developmental stage, when the lowest spikelet of the panicle was between 0 to 2 cm from the collar of the flag leaf, was selected for emasculation. (D) The spikelets from the tip and base of the panicle were trimmed leaving approximately 10 to 20 spikelets, and then their bristles were trimmed. (E) One third of each spikelet was removed using sharp scissors and then all three anthers from each spikelet of the panicle were removed using an ear pick and/or forceps. (F) The emasculated panicles were enclosed in glassine bags. (G) On the following day, the spikelets were checked and were dusted with pollen. (H) The pollination was repeated for two days. (I) The emasculated and pollinated panicles were then bagged until seed set. (J) Seed set was checked 10 to 12 days after pollination, if immature seed germination was required. (K) The seeds were allowed to mature and were harvested with care to avoid shattering.

For manual emasculation, magnifying lens, ear pick, scissors, and forceps were used (Fig 2A and 2B). The pointed end of the ear pick was rubbed with fine sand paper to make it sharp (Fig 2B). Spikelets which had emerged out of the leaf sheath were used for manual emasculation (Fig 2C). A number of spikelets, as per the need and efficiency of the scientist, were selected for hand emasculation and other spikelets were removed (Fig 2D). The top one third of each spikelet was cut off using sharp scissors (Fig 2E). Using the sharpened tip of the ear pick, three anthers of each spikelet were removed, without disturbing the white stigma. The emasculated spikelets were marked with a marker pen, to make sure all the spikelets in the panicle were emasculated. After emasculation, the panicles were bagged using glassine bags of appropriate sizes (Fig 2F), which were only removed during pollination and at the time of harvest. The following day, panicles were checked for any remaining anthers. If there were anthers coming out of any spikelet, the panicle was discarded from the crossing pipeline. Panicles without any live anthers were selected for pollination. Pollination was done by dusting with pollen on the second and third day (Fig 2G and 2H). The panicles were bagged again until the harvest (Fig 2I). Whenever necessary, immature seeds 10 to 12 days after pollination were harvested and grown in the plant growth medium (Fig 2J and 2K); otherwise the seeds were allowed to mature normally.

### Pollination

For pollination, fresh pollens were collected in a glassine bag. The pollens were sprinkled over the emasculated panicles on the third day of chemical emasculation or on the second day of manual emasculation. Before pollination, the emasculated spikelets were checked for any fresh anthers. Pollination was done once a day for 2 to 3 consecutive days. The panicles were placed inside glassine bags until the seeds set. Panicles were harvested individually when most of the seeds turned black indicating their physiological maturity.

### SSR marker and PCR verification

Simple sequence repeat (SSR) markers were developed to verify polymorphism between the A10.1 and PI 202407 accessions of *S*. *viridis*. The whole genome sequences were downloaded from NCBI SRA ID: SRP004296 for A10.1 (http://trace.ncbi.nlm.nih.gov/Traces/sra/?run=SRR072201) and NCBI SRA ID: SRP004291 for PI 202407 (http://trace.ncbi.nlm.nih.gov/Traces/sra/?run=SRR072180) on 2^nd^ May 2013. Several SSR regions between the two sequences were identified for alteration in size and one was selected for use. We verified the F_1_ crosses using SSR primer set with forward primer: 5´-AGCTTACAGTACATTAGGCAC-3´ and reverse primer: 5´-ATTTGGGTACAATCTAAGGGC-3´. The polymerase chain reaction (PCR) mixture consisted of 5 μL of 2x Terra buffer (Clontech Laboratories, Inc.), 0.07 μL Terra Taq (Terra PCR Direct Genotyping Kit [28]); 0.5 μL each of forward and reverse primers and final reaction volume was made 10 μL by adding double distilled water. A small piece of leaf of 0.5 to 1.0 mm size was used as the template instead of extracted and purified DNA. The PCR program consisted of initial denaturation at 98°C for 2 min, followed by 35 cycles of denaturation at 98°C for 10 sec, annealing at 60°C for 20 sec, and elongation at 68°C for 30 sec. The final elongation was performed at 68°C for 5 min. PCR products were electrophoresed on 2% agarose gel with 1x TAE buffer and visualized under UV light with SYBRSafe DNA gel stain (Invitrogen).

### Germination of F_1_ seeds in culture medium

#### a. Mature seeds

**Fig 3:**
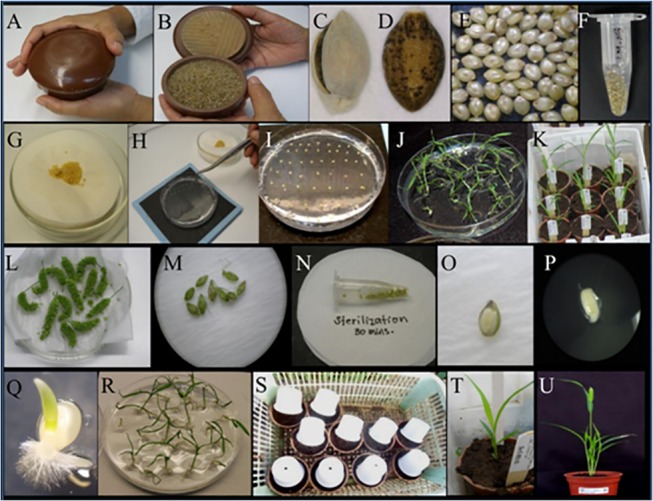
Seed germination in medium. (A, B) A dehuller was used to remove the seed coats of mature seeds. *S*. *viridis* seeds have two layers of seed coats, (C) outer seed coat and (D) inner seed coat. (E) Seeds after removal of both the seed coats look translucent white. (F) The dehulled seeds were washed with 70% ethanol and then with distilled water and were sterilized by incubating in a solution of 50% sodium hypochlorite containing 1 drop of Tween 20 (~2%) for 30 min. (G) The sterilized seeds were dried by blotting on filter paper. (H, I) The seeds were plated in MS medium with the embryo side facing upward. (J) All seeds germinated well in MS medium producing good root and shoot. (K) The seedlings after 10 days of plating were transplanted to the pots with soil and taken special care. (L) Immature panicles 10 to 12 days after pollination were selected for germination in MS medium. (M) Filled spikelets were chosen. (N) Selected spikelets were sterilized in 1% sodium hypochlorite solution with one drop of Tween 20 (~2%). (O) Immature embryos were dissected using sterilized scalpel and forceps, and (P) plated in MS medium. (Q) The immature embryos emerged into a seedling after 18 hrs of incubation in medium. (R) Small seedlings with well developed roots and shoots (10 to 14 days old after plating). (S) Seedlings were transplanted into pots with fine soil and covered with a translucent white cup for 2 days to allow the seedlings to adapt to the soil condition. (T) Seedlings from immature embryos could establish well in soil and (U) grew normally to maturity.

The seed coats of mature *S*. *viridis* were removed using a wooden dehuller (Fig 3A and 3B). Two layers of seed coats were removed to get the dehulled seeds (Fig 3C, 3D and 3E). Dehulled seeds were kept in 1.5 mL microtubes and rinsed with 70% ethanol, washed with distilled water and sterilized using 1mL of 50% sodium hypochlorite solution containing 1 drop of Tween 20 (~2%) and incubated in this solution for 30 min with continuous shaking. The sterilized seeds were then dried by blotting on filter paper (Fig 3G). These seeds were placed in 1x MS medium (pH = 5.8) gelled with gelrite (Sigma) with the embryo side facing upwards and incubated under light at 30°C (Fig 3H and 3I).

#### b. Immature seeds

Immature embryos were isolated from spikelets 10–12 days after pollination. The green spikelets were sterilized in 1 mL of 1% sodium hypochlorite solution containing 1 drop of Tween 20 (~2%). Glumes and palea of the sterilized spikelets were removed using forceps. Immature seeds were placed under the microscope (10X). The embryos were excised from the immature seeds using a sharp blade and forceps, and were placed in MS medium mentioned above. The embryos germinated into seedlings within 2 days. These were grown in media for 10 days before being transplanted to sterilized soil with appropriate fertilizers (Fig 3).

## Results

### (1) Selection of a suitable chemical gametocide

**Fig 4:**
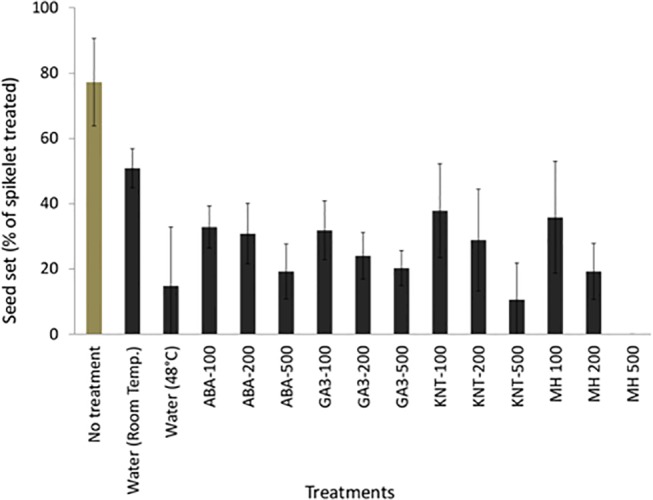
The effect of different chemical treatments on emasculation of *Setaria viridis*. The percentage of seed set in the panicles treated with different methods of emasculation. Ten plants with 50 spikelets per panicle were used for each treatment. Error bars show the standard deviation. All solutions were prepared in water. Room temperature water treatment and no treatment were used as controls.

We treated the panicles of *S*. *viridis* with 100, 200, and 500 μM solutions of ABA, GA3, KNT, and MH. We found that only the MH solutions of 500 μM and higher concentrations were able to suppress the seed set completely, hence MH was the most effective chemical gametocide that could be used for the chemical emasculation of *S*. *viridis* (Table 1). The panicles treated with water at 48°C had 15% seed set, whereas the water at room temperature reduced fertilization by 50%. The panicles without any treatment except trimming and uniform handling had 77% seed set (Fig 4). The panicles treated with 100, 200, and 500 μM of KNT had 38%, 29%, and 11% seed set, GA3 had 32%, 24%, and 20% seed set, ABA had 16%, 15%, and 10% seed set, and MH had 36%, 19%, and 0% seed set, respectively (Fig 4). The standard deviation of percentage of seed set was lower in treatments with higher concentrations of MH.

**Table 1:** The number of seed set in maleic hydrazide treated panicles with and without pollination.

Description	Not pollinated after emasculation	Pollinated after emasculation
No. of panicles emasculated	76	76
No. of panicles without seed set (%)	61 (80)	18 (24)
No. of panicles with seed set (%)	15 (20)	58 (76)
Total no. of spikelets treated	5568	3598
No. of spikelet with seed set (%)	150 (2.69)	787 (22)

### (2) Effectiveness of MH

**Fig 5:**
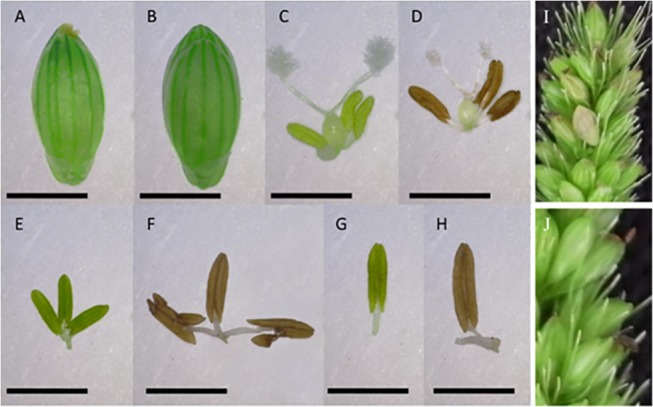
The effect of maleic hydrazide (MH) on the pre-anthesis anthers of *Setaria*. (A) Untreated control spikelet and (B) spikelet treated with 500 μM MH. (C and D) Androecium and gynoecium of control and MH-treated spikelets, respectively. (E and F) Anthers of control and MH-treated spikelets, respectively. (G and H) A representative anther of control and MH-treated spikelets, respectively. (I) In control panicles, mature seeds were observed in the spikelets. (J) Brown specks were observed as remnants of anther dehiscence in the MH-treated panicles and no seeds were set although the florets remained green. The scale bar represents 5 mm in A and B, 0.5 mm in C, D, E and F, and 0.75 mm in G and H; I and J are not to scale.

Emasculation was effective with the application of MH solutions of 500 μM and higher concentrations. The rate of seed set after pollination of the MH-emasculated panicles was significantly higher. A total of 76 plants were emasculated and set aside for seed set without pollination and another 76 plants were emasculated and pollinated three times. We observed that in the MH-treated but not pollinated plants, although 20% plants had seed set it was in only 3% of the total number of spikelets treated (Table 1). On the other hand, in the MH-treated and pollinated plants, 76% plants had seed set, which comprised 22% of the total spikelets. The seeds that emerged in the non-pollinated panicles could be due to the presence of spikelets which were too young at the time of treatment and the anthers that were active after the treatment. MH caused the browning of the anthers (Fig 5D, 5F and 5H), while the untreated anthers were green (Fig 5C, 5E and 5G). In the MH-treated spikelets, there were remains of brown specks on the tips which were observed as reminiscent of anther dehiscence. Effectiveness of higher concentrations of MH such as 1000 μM, 2000 μM and 5000 μM were also tested. No seeds were set in the case of the panicles treated with higher concentrations of MH even after cross pollination which means the higher concentrations of MH adversely affected the receptivity of the stigma (S2 Table).

Dipping of panicles in 500 μM MH solution for 2 min a day for 3 consecutive days caused agglutination of anthers. The stigmas were still white and receptive (Fig 5D). We observed 3% self-pollinated seeds in the emasculated stigmas and the seed set increased to 22% after pollination, confirming the efficiency of the MH in suppressing the pollen viability without adversely affecting the receptivity of the stigma (Table 1 and Fig 5).

### (3) Manual emasculation of *Setaria viridis*

We improvised the manual method to emasculate the small spikelets of *S*. *viridis* that are highly shattering. We used a magnifying glass to magnify the size of spikelets to work on (Fig 2). We trimmed the top one third of the spikelets and removed the anthers using a modified ear pick (Fig 2B). The seed set from the manual emasculation followed by pollination by dusting was up to 6%, which is low, but sufficient for small scale pollination studies and in the case where chemicals for emasculation are not readily available.

### (4) Genetic verification of the F_1_ plants with PCR using SSR marker

**Fig 6:**
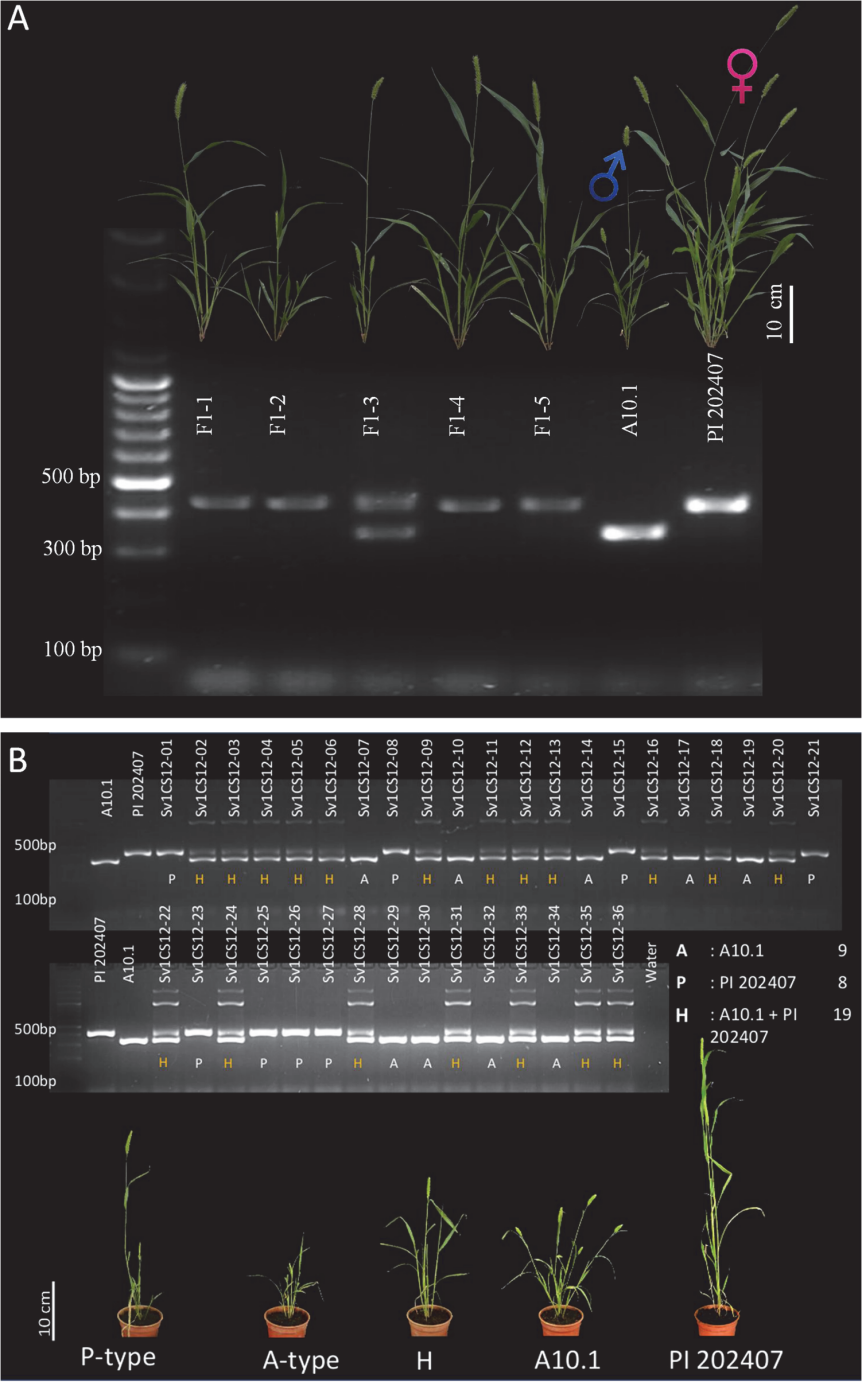
PCR verification of the crossed progenies using SSR markers. (A) Products of PCR using an SSR marker on F_1_ plants and the two parents (A10.1 and P1 202407). (B) PCR using the same SSR primer pair on the F_2_ plants obtained from self pollination of the F_1_ (F_1_-3, shown in Fig 6A), and phenotypes of the representative plants of parents A10.1 (A) and PI 202407 (P). Plants from F_2_ generation were labeled A-type, P-type and H-type (heterozygous having both A-type and P-type alleles) plants.

The F_1_ plants from the crosses between PI 202407 and A10.1 as well as from their reciprocal crosses were not easy to differentiate as the phenotypes of the two parents differed only slightly. Therefore, we developed polymorphic SSR primers to screen and verify the true crosses (Fig 6). With this primer set, the PCR amplicon of PI 202407 was about 50 bp more than the A10.1 amplicon (Fig 6A). It was difficult to calculate the exact numbers of base pairs in each amplicon due to the presence of several unidentified ‘n’ in the reference genome sequence in this region. The true F_1_ plants produced two PCR amplicons, one corresponding to the A10.1 parent ~370 bp and the other to the PI 202407 parent ~420 bp (Fig 6A). We screened the entire F_1_ population using this primer set. We also checked the segregation of the F_2_ population using the SSR marker. For example, in one of the F_2_ populations, the number of A-type (A10.1 parental type), P-type (PI 202407 parental type) and H (heterozygous, which had bands of both A-type and P-type) F_2_ plants was 9, 8 and 19 confirming to 1:1:2 (X^2^ = 0.167 with 2 degree of freedom; the two tailed p value = 0.9200) indicating that this population followed the standard Mendelian segregation for the F_2_ generation (Fig 6B). Thus, we confirmed the reliability of the marker and the method of crossing.

### (5) Generation advancement

To screen a large number of mutant populations and develop homozygous lines, we needed to further shorten the time to produce F_2_ seeds. We opted to use immature embryos to grow in MS media so that we would not need to wait until maturity. Crosses with mutants often produced less vigorous embryos, which called for embryo rescue of immature F_1_ seeds, which were grown in culture medium instead of waiting for them to physiologically mature. The F_1_ seeds from the chemical emasculation were treated more sensitively than the seeds from the normal seed sets. The mature seeds were dehulled using a wooden dehuller when the number of seeds was high enough, sterilized using ethanol, washed with distilled and autoclaved water, and soaked in a solution of 50% sodium hypochlorite and ~2% Tween 20. The seeds were grown in MS culture medium and kept under continuous light at 30°C. Mature seeds were grown directly in medium or in soil. In the case of immature seeds, embryos were first isolated (Fig 3O) and grown in medium (Fig 3P). Seedlings that were 10 to 14 days old were transplanted directly into the soil and acclimatized in a controlled environment for 2 days (Fig 3S).

Germination of seeds in plant growth medium was preferred when the numbers of seeds were few and the chances of germination were low if directly sown in soil. Seeds from crosses, mutants and distant crosses were germinated in culture medium. On average, the germination rate of these seeds in medium was 83% (S1 Table). Performing embryo rescue of the immature seeds and growing them in medium saved the time the seeds took to mature normally and the time for breaking dormancy, hence, accelerating generation advancement. *S*. *viridis* normally completes its seed to seed cycle in 60 to 70 days. Embryo rescue can reduce 20 to 30 days per life cycle, thus making it faster to obtain the next generation plants.

## Discussion

Manual and hot water emasculations are effective in many plant species. In *S*. *viridis*, manual emasculation is a constraint due to the small size of its floral parts and shattering of spikelets. We optimized the manual emasculation technique, which can be used when the chemicals are not readily available and when the number of crosses required are few. The flaw of the warm water treatment is the large number of selfed seeds. The successful use of MH as chemical emasculant at a concentration of 100 μM from 25 days after sowing till the cessation of flowering was reported for coriander [27]. Also, there have been some reports on the effect of MH on culture medium used for both animals, and plants [29]. Being a pyridazine compound, MH is known as a clastogenic agent in plants and is known to inhibit the synthesis of nucleic acids and proteins [30, 31]. The application of MH in *Vicia faba* was reported to induce chromosome aberrations and sister chromatid exchanges in the root meristems [32, 33]. Further, it was found to have mutagenic and carcinogenic effects on cell cultures and animals, but no effects were reported in humans [34]. Although considered a weak cytogenetic or mutagenic chemical and ranked as a class 4 chemical [35], we observed the necessary handling precautions using gloves and a breathing mask. With some caution and safety equipment during application, MH can be used to effectively make the anthers of *S*. *viridis* sterile.

Chemical emasculation reduces labor-intensive manual work, as one person can trim and emasculate up to 30 panicles in half a day. On the other hand, the chemical induced male sterility gives the breeder the freedom to use or change the parents as per need and crossing for fertility restoration is not needed [36]. The stigmas remained receptive after MH treatment and an average seed set of 22% was achieved. This is good enough to develop a hybrid population for research purposes. The F_1_ plants appeared healthy, grew normally and produced seeds like the parental lines, which precludes the possibility of the mutagenic effect of MH in *Setaria viridis* at the given concentration.

Growing of both immature and mature seeds in culture medium renders the need for breaking seed dormancy obsolete, improves germination and accelerates the breeding cycle, which saves time for the scientist to obtain new generations.

This is the first comprehensive study to test effect of various chemicals on pollen sterility of *S*. *viridis*. MH was identified to be the most effective chemical gametocide and its concentration was optimized at 500 μM for *S*. *viridis* emasculation. The chemical emasculation technique developed in this study will greatly enhance the use of *Setaria* for research on C_4_ photosynthesis, weeds, and biofuel. The use of optimized culture medium for germinating mature and immature seeds of *S*. *viridis* reduces the time for advancing generations which is an important step for those involved in basic research of genetic inheritance.

## Supporting Information

S1 Protocol Protocols for chemical and manual emasculation of green foxtail (*Setaria viridis*) flowers and growing of immature and mature seeds in plant culture medium to rescue sensitive seeds as well as to accelerate breeding cycle. (DOCX)

S1 Table Germination percentage of *S. viridis* seeds in plant culture medium. Seeds of different generations and background that needed extra care were grown in culture medium.
(XLSX)

S2 Table The number of seed set in panicles of *Setaria viridis* treated with different doses of maleic hydrazide (MH). (XLSX)
