# Supplementary material for: The Use of Maleic Hydrazide for Effective Hybridization of Setaria viridis
Source: PLoS One. 2015 Apr 24;10(4):e0125092. doi: 10.1371/journal.pone.0125092 (PMC4409208; doi:10.1371/journal.pone.0125092)
Supplement: S1 Protocol — (DOCX) [file pone.0125092.s001.docx]

**S1_Protocol.** Protocols for chemical and manual emasculation of green foxtail (*Setaria viridis*) flowers and growing of immature and mature seeds in plant culture medium to rescue sensitive seeds as well as to accelerate breeding cycle.

1. **Cultivation of *Setaria viridis***
2. *Setaria viridis* was grown in greenhouse conditions in International Rice Research Institute (IRRI) Los Banos (14^o^ 11” N, 121^o^ 15” E). The ranges of diurnal and nocturnal temperatures were 28 to 33^o^C and 24 to 27^o^C, respectively; with an average 12 hr light/12 hr dark.
3. Soil was fertilized with nitrogen, phosphorous and potassium (NPK) at the rate of 10 g NPK for 100 kg of soil and put into 100 cm^3^ cups.
4. The soil was moistened by lightly spraying with water.
5. The seeds were sown in the center of the cup and covered thinly with fine soil.
6. The plants were watered twice a day (i.e. 07:00 and 17:00 h) for the first week and once a day thereafter.
7. Plants were fertilized every 7 days with 0.6 g of urea per pot.
8. Anthesis in *S.viridis* was observed between 18 and 25 days after sowing (DAS) when grown in the above conditions.
9. **Panicle preparation**
10. Panicles that had newly emerged (0 to 2 cm from the collar) from their flag leaves were selected.
11. Individual spikelets in the panicle were checked for evidence of anther dehiscence using a magnifying glass.
12. The spikelets were checked to ensure that they had not opened or dehisced (anthers are seen as small yellow to brown specs on the tip of the spikelet).
13. Spikelets at the tip and base of the panicle were trimmed using sharp scissors or forceps.
14. The bristles were trimmed.
15. Approximately 50 spikelets per panicle were retained for chemical or hot water treatment and 10 to 20 spikelets per panicle for manual emasculation.
16. **Emasculations**
17. **Manual emasculation**
    1. An illuminated ear pick was rubbed with fine sand paper to make it sharp and pointed.
    2. The illuminated ear pick was cleaned with 70% ethanol.
    3. An illuminated desk magnifying lens was used to view the spikelets.
    4. One third of a spikelet was trimmed from its tip using sharp scissors.
    5. The three anthers inside spikelet were carefully removed using ear pick and/or forceps without touching or damaging the white stigma.
    6. The emasculated spikelet was marked with ink and this was repeated until all spikelets were emasculated.
18. **Chemical emasculation**
    1. 500 µM Maleic hydrazide (MH) solution was freshly prepared; (19.6 mg MH was dissolved in 100 mL of distilled water, pH = 7.0 adjusted using 1 N NaOH and then the volume was adjusted to 350 mL with distilled water). The solution was prepared inside a fume hood.
    2. The solution was dispensed into 50 mL falcon tubes for emasculation and stored at 4°C until use. The half-life of MH in water is 30 days at pH 7.0. We used the solution within a week of preparation.
    3. The trimmed panicles were immersed into the MH solution for two minutes (we wore gloves and mask for safety).
    4. The panicles were gently rubbed with tissue paper, left to dry, then bagged and labeled.
    5. This was repeated for three consecutive days from 8:00 to 10:00 am.
    6. The panicle was kept bagged until seeds were harvested.
19. **Quality control**
    1. **Manual or chemical emasculation**
    2. The day after emasculation, the panicles were checked for any signs of dehiscence.
    3. Panicles with evidence of anther dehiscence were discarded.
    4. The panicles were also checked for their healthy physical condition. The panicles with signs of mechanical damage, such as browning of spikelets, were discarded.
20. **Cross pollination**
    1. On the third day of emasculation, the spikelets were checked for opening and stigma receptivity (presence of white feathery stigma).
    2. Pollination was performed by dusting pollen or panicle pairing.

**2.1. Pollen dusting**

Pollen was collected from the panicle of the selected plant by wiping with glassine paper and then dusted over the emasculated and control spikelets.

**2.2. Panicle pairing**

Panicles that would dehisce the next day were attached to the emasculated panicle. The paired panicles were secured using a paper clip and then covered with a glassine bag.

1. **Harvesting of seeds**
   1. Normally, seeds were harvested about 3 weeks after pollination when the seeds had turned black, except in manually emasculated seeds where the seeds were without the black seed coat. Immature seeds were harvested 10-12 days after pollination.
   2. The harvested mature seeds were sun-dried for about three days.
   3. The dried seeds were stored at 4°C or germinated in the culture medium.

**Germination in culture medium**

**Harvesting and sterilization for germination in medium**

1. Spikelets were harvested 10-12 days after anthesis or pollination.

2. Using sterile forceps immature seeds were dehulled.

3. Seeds were put into 1.5 mL microtube containing 70% ethanol and soaked for 1 min for sterilization.

4. The seeds were then washed with sterile distilled water.

5. The seeds were washed again with 1% (use 50% for mature seeds) sodium hypochlorite containing 1 drop of Tween20 (~2 %) solution for 5 min with continuous shaking. The solution was then discarded and the seeds were washed at least 5 times with sterile distilled water, or until the water was clear.

6. The immature embryos were dissected under a stereo dissection microscope with a sterilized scalpel and forceps.

7. The immature embryos were placed in MS medium (Murashige and Skoog, 1962) with the scutellum side facing down. The plates were sealed with micropore tape and incubated at 30°C under continuous light. Root and shoot of the seedlings started to emerge the next day.

8. After 10 days or when the roots and shoots are well developed, the seedlings were transferred to autoclaved soil with appropriate nutrition and watered lightly.

**Reference**

Murashige T, Skoog F. A revised medium for rapid growth and bio-assays with tobacco tissue cultures. Physiol Plant 1962; 15: 473–497.
